# DNMT1 involved in the analgesic effect of folic acid on gastric hypersensitivity through downregulating ASIC1 in adult offspring rats with prenatal maternal stress

**DOI:** 10.1111/cns.14131

**Published:** 2023-02-27

**Authors:** Hong‐Jun Wang, Fu‐Chao Zhang, Timothy W. Xu, Yu‐Cheng Xu, Yuan‐Qing Tian, Yan‐Yan Wu, Ji‐Tian Xu, Shufen Hu, Guang‐Yin Xu

**Affiliations:** ^1^ Jiangsu Key Laboratory of Neuropsychiatric Diseases and Institute of Neuroscience Soochow University Suzhou Jiangsu China; ^2^ Jiangsu Key Laboratory of Anesthesiology & Jiangsu Key Laboratory of Anesthesia and Analgesia Application Technology Xuzhou Medical University Xuzhou China; ^3^ Suzhou Academy of Xi'an Jiaotong University Suzhou China; ^4^ Department of Physiology and Neurobiology College of Basic Medical Sciences, Zhengzhou University Zhengzhou China

**Keywords:** acid‐sensing ion channel, DNA methyltransferase, dorsal root ganglion, folic acid, functional dyspepsia, gastric hypersensitivity

## Abstract

**Aims:**

Gastric hypersensitivity (GHS) is a characteristic pathogenesis of functional dyspepsia (FD). DNA methyltransferase 1 (DNMT1) and acid‐sensing ion channel 1 (ASIC1) are associated with GHS induced by prenatal maternal stress (PMS). The aim of this study was to investigate the mechanism of DNMT1 mediating the analgesic effect of folic acid (FA) on PMS‐induced GHS.

**Methods:**

GHS was quantified by electromyogram recordings. The expression of DNMT1, DNMT3a, DNMT3b, and ASIC1 were detected by western blot, RT‐PCR, and double‐immunofluorescence. Neuronal excitability and proton‐elicited currents of dorsal root ganglion (DRG) neurons were determined by whole‐cell patch clamp recordings.

**Results:**

The expression of DNMT1, but not DNMT3a or DNMT3b, was decreased in DRGs of PMS rats. FA alleviated PMS‐induced GHS and hyperexcitability of DRG neurons. FA also increased DNMT1 and decreased ASIC1 expression and sensitivity. Intrathecal injection of DNMT1 inhibitor DC‐517 attenuated the effect of FA on GHS alleviation and ASIC1 downregulation. Overexpression of DNMT1 with lentivirus not only rescued ASIC1 upregulation and hypersensitivity, but also alleviated GHS and hyperexcitability of DRG neurons induced by PMS.

**Conclusions:**

These results indicate that increased DNMT1 contributes to the analgesic effect of FA on PMS‐induced GHS by reducing ASIC1 expression and sensitivity.

## Graphical abstract

The present study has shown that FA alleviates PMS‐induced GHS through increasing DNMT1 and decreasing ASIC1, respectively, and blocking DNMT1 attenuate the effect of FA on GHS and ASIC1 expression. These findings suggest that DNMT1 mediates the analgesic effect of FA on GHS through downregulating ASIC1 and may be a therapeutic target for the treatment of visceral pain.

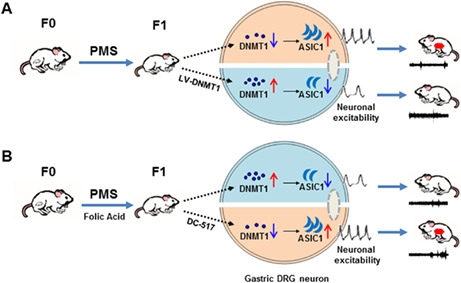

## 1 INTRODUCTION

Functional dyspepsia (FD) is a common clinical gastrointestinal disease with a high prevalence worldwide.^1^, ^2^ Gastric hypersensitivity (GHS) responding to gastric distention represents a cardinal pathophysiological change in FD.^3^, ^4^, ^5^ However, the underlying mechanisms are not fully understood. Adverse early life events, such as severe psychological stress, are established risk factors for the development of functional bowel disorders in adulthood.^6^, ^7^ Our previous study demonstrated that prenatal maternal stress (PMS) induced GHS through enhanced acid‐sensing ion channel 1 (ASIC1) demethylation.^8^ Others also reported that the pathogenesis of visceral pain involves dynamic changes in the epigenetic markers and gene expression.^9^, ^10^, ^11^ As a result, epigenetic regulation may represent a novel mechanism of FD, and deciphering the mechanisms underlying is crucial for the treatment of visceral pain.

DNA methylation is a type of epigenetic modification and is catalyzed primarily by a family of DNA methyltransferases (DNMTs), including DNMT1, DNMT3a, and DNMT3b.^12^, ^13^ Epigenetic regulation is not static as it can be altered by environmental stimuli.^14^, ^15^ The contribution of DNMT1 and DNMT3a‐triggered DNA methylation to neuropathic pain has been reported.^16^, ^17^, ^18^ The mRNA of Dnmt1, but not Dnmt3a and Dnmt3b, is downregulated in dorsal root ganglion (DRG) of PMS rats, indicating an involvement of DNMT1 in the development of FD.^8^ Nevertheless, the role of DNMT1 in the pathogenesis of PMS‐induced GHS has not yet been validated.

Folic acid (FA) is a major component of one‐carbon metabolism that plays a critical role in the nervous system.^19^, ^20^ Accumulating evidence links disorders of one‐carbon metabolism to neurodegenerative and neuropsychiatric diseases, including Alzheimer's dementia and depression.^21^, ^22^ An adequate amount of FA is required during pregnancy for the rapid rate of tissue growth.^23^ FA supplementation during the periconceptional period reduces the prevalence of neural tube defects.^24^, ^25^ Moreover, systemic administration of FA improves axonal regeneration through DNA methylation.^26^ FA also acts through the elevation of DNMT activity to increase neuronal differentiation in neural stem cells.^27^ The mandatory fortification with FA might alter the expression of DNMT1 in cervical carcinogenesis.^28^ Therefore, FA may be useful to promote healing in neural tissues. However, whether FA supplementation alleviates PMS‐induced GHS has not yet been explored.

We have established a PMS‐induced FD model and confirmed the decrease of DNMT1 and increase of ASIC1 in PMS rats.^8^ In the present study, we aimed to further investigate the mechanism of DNMT1 in the analgesic effect of FA on GHS. Our findings indicated that FA is required to relieve GHS, possibly through elevating DNMT1, then inhibiting ASIC1 and neuronal excitability. Treatments with DNMT1 inhibitor blocked the analgesic effect of FA on GHS. Moreover, overexpression of DNMT1 reduces neuronal hyperexcitability and GHS. Therefore, FA and DNMT1 provide a potential treatment strategy for visceral pain.

## 2 MATERIALS AND METHODS

### 2.1 Animals

Experiments were performed on pregnant Sprague–Dawley (SD) rats and their adult male offspring. The onset of pregnancy was confirmed by the presence of a vaginal plug, which was defined as gestational day 1 as described previously.^29^ All experiments were approved by the Institutional Animal Care and Use Committee of Soochow University. The care and handling of all rats followed the guidelines of the International Association for the Study of Pain.

### 2.2 PMS procedure

Prenatal maternal stress pregnant dams were subjected to a heterotypic intermittent stress protocol, as described previously.^30^, ^31^ Age‐matched control (CON) pregnant dams underwent sham stress, and their male offspring were designated control rats.

### 2.3 Drugs and administration

Folic acid (80 μg/kg, Sigma) or normal saline (NS) was intraperitoneally injected from gestational day 7 to delivery according to the changed body weight in PMS rats. To determine the role of DNMT1 in the analgesic effect of FA, DC‐517, solubilized in dimethyl sulfoxide (DMSO), was used to inhibit DNMT1. The intrathecal injection of DC‐517 or DMSO was adopted once a day for 7 days consecutively in FA‐treated PMS rats at the age of 6 weeks. On the other hand, lentivirus (LV‐NC) or lentivirus expressing full‐length Dnmt1 (LV‐DNMT1) was used as the negative control or to overexpress DNMT1 (Shanghai Gene Chem Co. Ltd). The estimated titer of the concentrated LV‐NC or LV‐DNMT1 was 1.25 × 10^8^ transducing units per milliliter (TU/mL). A volume of 10 μL of LV‐NC or LV‐DNMT1 was intrathecally injected in PMS rats at the age of 5 weeks. After 2 weeks, EMG recordings were applied to determine GHS, and whole‐cell patch‐clamp recordings were conducted to detect the excitability of DRG neurons.

### 2.4 EMG recordings

The procedure for the insertion of the gastric distension (GD) balloon and recording EMG activities were conducted as described previously.^6^ Gastric hypersensitivity (GHS) to balloon distension was measured by an observer in a blinded manner. Rats received a series of 20 s graded GD: 20, 40, 60, 80, and 100 mmHg, with 2 min intervals between distensions. A Biopac electromyogram (EMG)‐150C amplifier (Biopac Systems, Inc) continuously recorded EMG. The traces were analyzed using Acknowledge (Biopac Systems, Inc). The response to GD was defined as an increase in EMG activity above baseline during the 20 s GD period. Data were reported as the area under the curve (AUC) of the integrated EMG after baseline subtraction.

### 2.5 Labeling of gastric‐specific DRG neurons

Gastric‐specific DRG neurons were labeled with 1,1'‐dioleyl‐3,3,3',3‐tetramethylindocarbocyanine methanesulfonate (Dil, 50 mg in 1 mL methanol, Invitrogen, California, USA) in the stomach wall. Ten days later, T7–T10 DRGs were dissected for whole‐cell patch clamp recordings or immunofluorescence.^32^

### 2.6 Whole‐cell patch clamp recordings

The changes in neuronal excitability and proton‐elicited currents were determined as described previously.^33^ Briefly, T7–T10 DRGs were dissected out and transferred to an ice‐old and oxygenated fresh dissecting solution Then, the DRG neurons were dissociated as described previously.^34^ Dissecting solution (in mmol/L): 130 NaCl, 5 KCl, 2 KH_2_PO_4_, 1.5 CaCl_2_, 6 MgSO_4_, 10 glucose, 10 HEPES, pH 7.2, osmotic pressure 305 mOsm. Patch clamp recording external solutions (in mmol/L): 130 NaCl, 5 KCl, 2 KH_2_PO_4_, 2.5 CaCl_2_, 1 MgCl_2_, 10 HEPES, 10 glucose, pH adjusted to 7.2, NaOH regulation, osmotic pressure 295–300 mOsm. The recording internal solution (mmol/L): 140 potassium gluconate, 10 NaCl, 10 HEPES, 10 glucose, 5 EGTA, 1 CaCl_2_, pH 7.25, KOH regulation; 292 mOsm permeability. The enzyme solution containing collagenase D (1.8–2.0 mg/mL; Roche Diagnostics) and trypsin (1.2–1.5 mg/mL; Amresco, USA). After a single‐cell suspension was obtained, cells were plated onto acid‐cleaned glass coverslips. The coverslips containing adherent DRG neurons were placed in a small recording chamber and attached to the stage of an inverting microscope (Olympus IX71, Olympus). DiI‐labeled neurons were identified by fluorescence under a fluorescence microscope (Olympus IX71, Olympus). Recording pipettes were pulled from borosilicate glass tubing using a horizontal puller (P‐97, Sutter Instruments). Unless indicated, patch clamp pipettes had a resistance of 4–7 M when filled with the pipette solution. The voltage was clamped at −60 mV by a HEKA EPC10 patch‐clamp amplifier (HEKA Electronik GmBH; Germany). Whole‐cell current and voltage were recorded with a HEKA EPC10 patch‐clamp amplifier, and data were acquired and stored on a computer for later analysis with FitMaster (HEKA Electronik GmBH; Germany).

### 2.7 Real‐time polymerase chain reaction (RT‐PCR)

Total RNA was extracted from T7–T10 DRGs using TRIzol reagent (Invitrogen), and cDNA was synthesized using EasyScript First‐Strand cDNA Synthesis SuperMix kit (Transgen Biotech), according to the manufacturer's instructions. The primer sequences used in this study are listed in Table 1. Negative control reactions did not contain the cDNA temple. The relative expression levels of the target were calculated using the comparative 2^−∆∆Ct^ method and normalized to that of GAPDH.

**TABLE 1:** Oligonucleotides used in the study.

Primers	Sequences (5′–3′)
*Dnmt1‐*F	TACGCAAGGTTTGAGTCCCC
*Dnmt1‐*R *Dnmt3a‐*F *Dnmt3a‐*R	CCCAGTCGGTAGACAACACC GAGGGAACTGAGACCCCAC CTGGAAGGTGAGTCTTGGCA
*Dnmt3b‐*F	CATAAGTCGAAGGTGCGTCGT
*Dnmt3b‐*R	ACTTTTGTTCTCGCGTCTCCT
*Gapdh*‐F	TGGAGTCTACTGGCGTCTT
*Gapdh*‐R	TGTCATATTTCTCGTGGTTCA

### 2.8 Western blot

Western blot analyses with T7–T10 DRGs were performed as described previously.^32^ Nitrocellulose membranes were probed with primary antibodies: mouse anti‐DNMT1 (1:1000, Abcam), rabbit anti‐DNMT3a (1:1000, Abcam), rabbit anti‐DNMT3b (1:1000, Abcam), rabbit anti‐ASIC1 (1:1000, Alomone Labs), and rabbit anti‐GAPDH (1:1000; Hangzhou Goodhere Biotechnology) at 4°C overnight. Subsequently, the membranes were incubated with anti‐rabbit horseradish peroxidase (HRP)‐conjugated (1:2000, Multi Sciences Biotech Co.) or anti‐mouse HRP‐conjugated secondary antibody (1:4000, Sigma) at room temperature for 1 h. The immunoreactive bands were detected with ECL reagents using Image J software on a gel imaging system (Bio‐Rad).

### 2.9 Immunofluorescence

To detect the co‐expression of DNMT1 and ASIC1 in gastric DRG neurons, animals were deeply anesthetized and then perfused transcardially with 4% paraformaldehyde (PFA). The cryosections of T7–T10 DRGs were incubated with primary antibodies DNMT1 (1:100, Abcam) and AISC1 (1:50, Alomone Labs) at 4°C overnight. Then, the sections were incubated with a mixture of Alexa Fluor 405 Goat Anti‐Mouse (1:100, Abcam) and Alexa Fluor 488 Donkey Anti‐Rabbit (1:100, Thermo Fisher Scientific). The controls included the omission of the first and/or the second primary antibodies. The stained sections were viewed under a fluorescence microscope (Leica, Germany).

### 2.10 Rotarod test

Rotarod test was performed as described previously.^35^, ^36^ The rats were placed on a rotating rod with a non‐slippery surface motionless for 5 min to adapt to the rotarod apparatus. The time for which an animal could remain on the rotating rod was recorded using an accelerating rotarod. During the procedure on 3 consecutive days, the speed of the rod rotation increased gradually from 5 to 25 rpm over the course of 5 min. The rats stayed on the rod until falling off, which was measured using a timing device.

### 2.11 Statistics

Statistical analysis was conducted using OriginPro 8 (Origin Lab) and Prism 6 (GraphPad) software. Normal distribution of data was analyzed using the Kolmogorov–Smirnov test. Differences in groups with normally distributed data were analyzed using the two‐sample *t*‐test, two‐way repeated‐measures analysis of variance (ANOVA); data that do not exhibit a normal distribution were analyzed using Mann–Whitney test. All data were expressed as means ± standard error of the mean (SEM). A *p*‐value <0.05 indicated a statistically significant difference.

## 3 RESULTS

### 3.1 PMS induces GHS and decreases DNMT1 expression in DRGs

**FIGURE 1:**
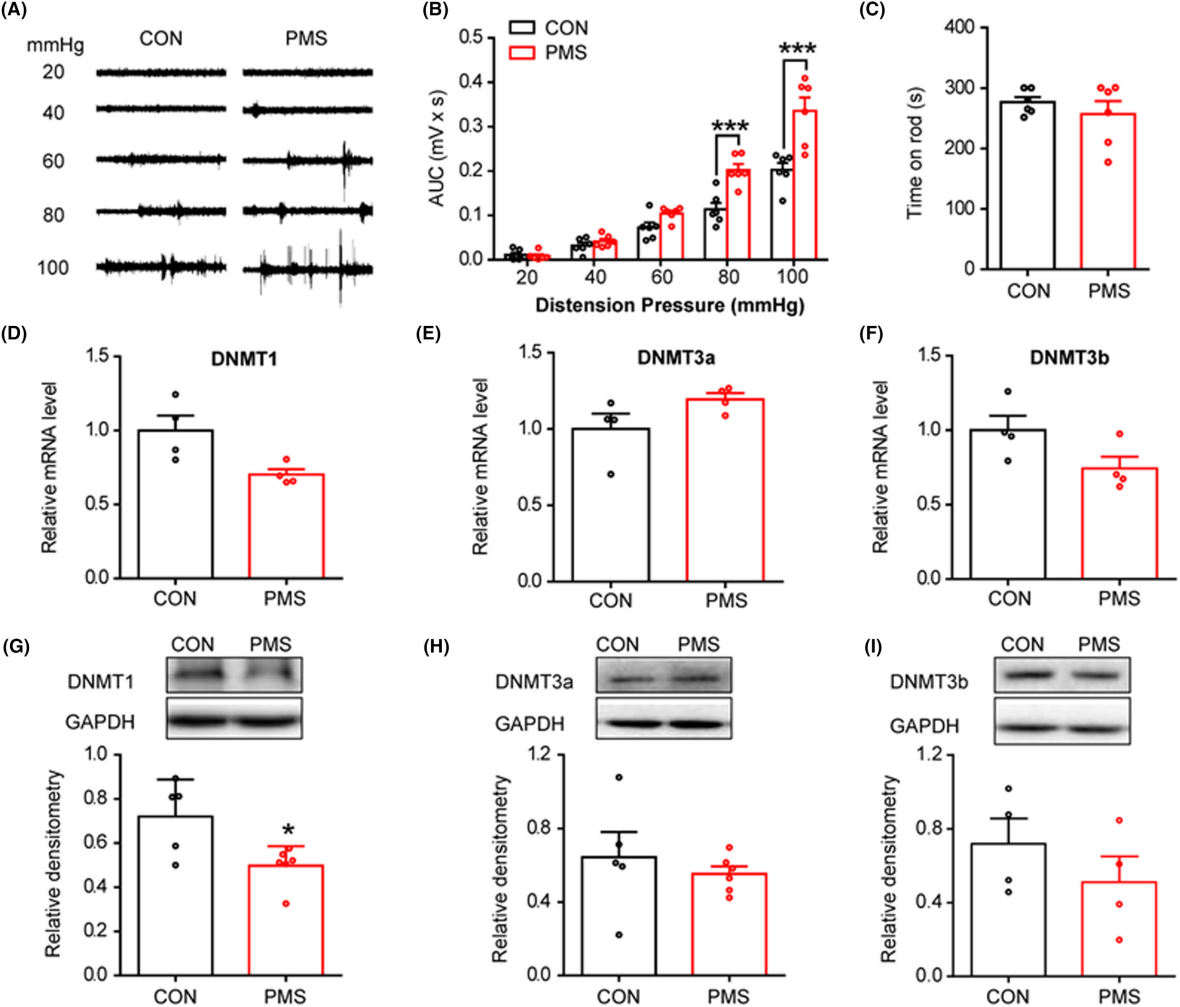
PMS induces GHS and decreases DNMT1 expression. (A) GHS induced by PMS at the age of 7 weeks. EMG recordings were applied to grade GD at 20, 40, 60, 80, and 100 mmHg in control and PMS rats. (B) The AUC from PMS rats was significantly higher than controls at distension pressures of 80 and 100 mmHg (*n* = 6 rats for each group, ***p < 0.001 vs. CON, two‐way repeated‐measures ANOVA). (C) PMS treatment did not affect the time for rats to stay on the rod (*n* = 6 rats for each group, *p* > 0.05 vs. CON, two‐sample *t*‐test). (D–F) RT‐PCR assays showed that the mRNA expression of Dnmt1, Dnmt3a, and Dnmt3b was not altered (*n* = 4 rats for each group, *p* > 0.05 vs. CON, Mann–Whitney test). (G) Western blot assays demonstrated that DNMT1 protein expression was significantly decreased in T7–T10 DRGs of PMS rats (*n* = 5 and 6 rats for CON and PMS group, respectively, *p < 0.05 vs. CON, two‐sample *t*‐test). (H and I) DNMT3a (*n* = 5 and 6 rats for CON and PMS group, respectively) and DNMT3b (*n* = 4 rats for each group) protein expression was not changed in T7–T10 DRGs of PMS rats (*p* > 0.05 vs. CON, two‐sample *t*‐test).

First, we confirmed that PMS could induce GHS, which was consistent with our previous study. GHS was assessed by measuring the viscerosomatic responses to graded GD using EMG. The gastric balloon and electrodes were implanted at the age of 6 weeks. One week after recovery from surgery, GD‐induced painful responses were recorded (Figure 1A). The adult offspring with PMS were more sensitive to GD, as evident by AUC. The results showed significant effects at the distension pressures of 80 and 100 mmHg (Figure 1B, *n* = 6 rats for each group, ***p < 0.001 compared to CON, two‐way repeated‐measures ANOVA). The motor function detected by rotarod was not affected (Figure 1C, *n* = 6 rats for each group, *p* > 0.05 compared to CON, two‐sample *t*‐test).

To determine whether an epigenetic mechanism, such as DNA methylation contributes to PMS‐induced GHS, we detected the potential changes of DNMTs. There was no change in the mRNA level of Dnmt1, Dnmt3a, and Dnmt3b (Figure 1D–F, *n* = 4 rats for each group, *p* > 0.05 compared to CON, Mann–Whitney test). However, the protein level of DNMT1 was decreased (Figure 1G, *n* = 5 and 6 rats for CON and PMS group, respectively, *p < 0.05 compared to CON, two‐sample *t*‐test), while no change was detected in DNMT3a (*n* = 5 and 6 rats for CON and PMS group, respectively) and DNMT3b (*n* = 4) (Figure 1H,I, *p* > 0.05 compared to CON, two‐sample *t*‐test or Mann–Whitney test). These data suggested that DNMT1 was significantly downregulated at the protein levels in PMS rats. Therefore, the present study mainly focused on the role of DNMT1 in PMS‐induced GHS.

### 3.2 FA treatment reverses GHS and enhances DNMT1 expression in T7–T10 DRGs of PMS rats

**FIGURE 2:**
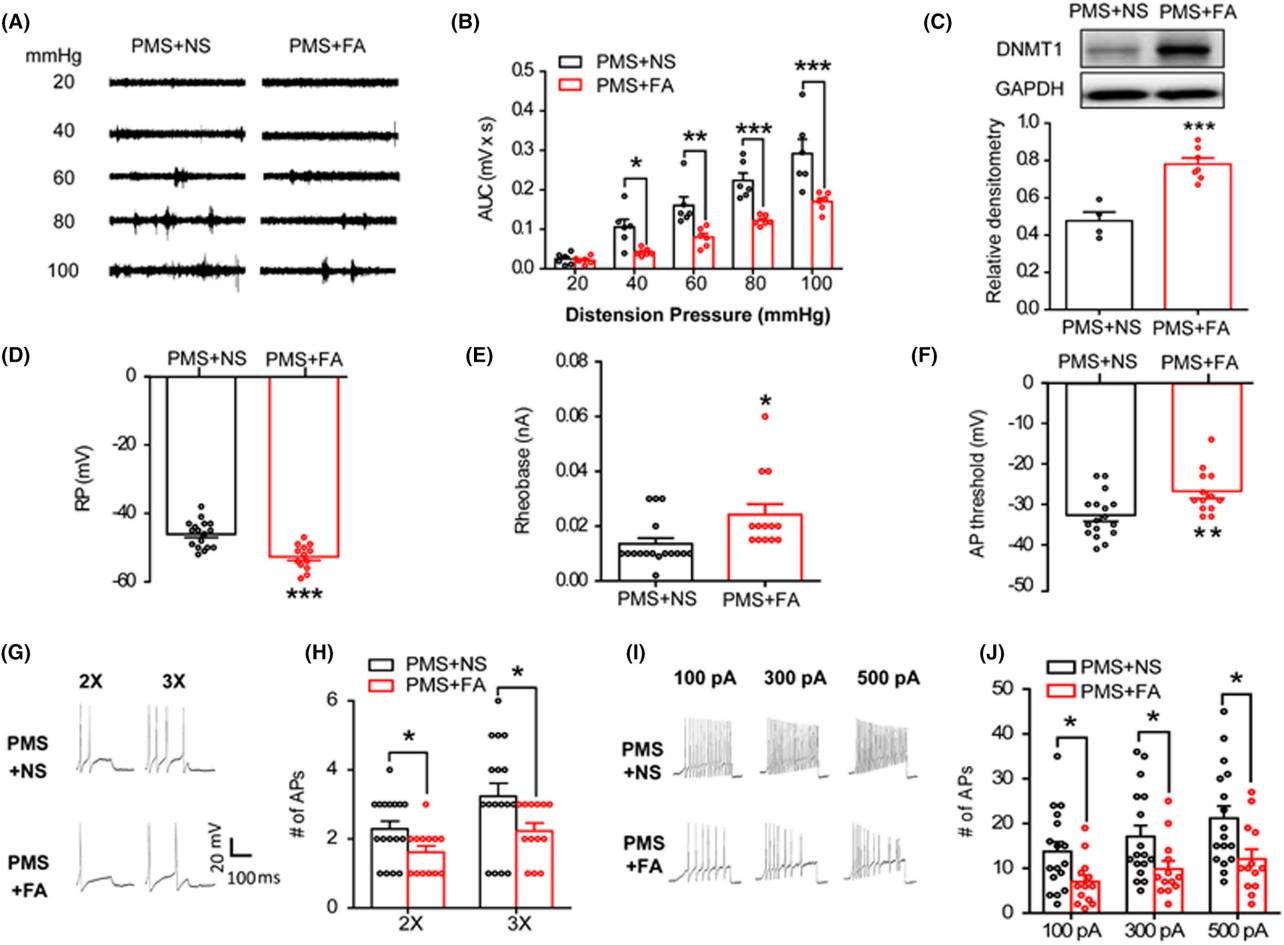
FA treatment alleviates GHS, enhances DNMT1 expression, and reverses neuronal hyperexcitability in PMS rats. (A) Representative EMG recordings from PMS + NS and PMS + FA rats responding to graded GD at 20, 40, 60, 80, and 100 mmHg at PND49. (B) FA at a dose of 80 μg /kg dramatically suppressed the AUC value at 40, 60, 80, and 100 mmHg GD pressures (*n* = 6 rats for each group, *p < 0.05, **p < 0.01, ***p < 0.001 vs. PMS + NS, two‐way repeated‐measures ANOVA). (C) Western blot results showed that FA treatment reversed DMNT1 protein expression in T7–10 DRGs of PMS rats (*n* = 4 and 7 rats for NS and FA group, respectively, ***p < 0.001 vs. PMS + NS, two‐sample *t*‐test). (D) Intraperitoneal injection of FA (80 μg/kg) markedly hyperpolarized RP (NS, *n* = 17 cells; FA, *n* = 13 cells, ***p < 0.001 vs. NS, two‐sample *t*‐test). (E) Administration of FA remarkably increased rheobase (NS, *n* = 17 cells; FA, *n* = 13 cells, *p < 0.05 vs. NS, two‐sample *t*‐test). (F) FA treatment significantly increased AP thresholds (NS, *n* = 17 cells; FA, *n* = 13 cells, **p < 0.01 vs. NS, two‐sample *t*‐test). (G) Examples of APs evoked by 2X and 3X rheobase current stimulation from NS (upper) and FA‐treated (bottom) rats. (H) Bar graph demonstrated a significant decrease in the numbers of APs evoked by 2X and 3X rheobase current stimulation in FA‐treated PMS rats (NS, *n* = 17 cells; FA, *n* = 13 cells, *p < 0.05, vs. NS, two‐sample *t*‐test). (I) Examples of APs evoked by 100, 300, and 500 pA ramp current stimulation from NS (upper) and FA‐treated (bottom) rats. (J) Bar graph showed a significant decrease in the numbers of APs evoked by 100, 300, and 500 pA ramp current stimulation in FA‐treated PMS rats (NS, *n* = 17 cells; FA, *n* = 13 cells, *p < 0.05, vs. NS, two‐sample *t*‐test).

As a cofactor in a multitude of single‐carbon transfer reactions, FA has a direct influence on epigenetic maintenance.^19^ Reduced FA is linked to a variety of clinical conditions, such as neurological and cardiovascular diseases.^22^ However, whether FA is involved in the development of PMS‐induced GHS is unclear. In the present study, FA (80 μg/kg) or NS was administered by intraperitoneal injection from gestational day 7 to delivery in PMS rats. At the age of 7 weeks, EMG was recorded in response to graded GD. FA treatment resulted in a dramatic reduction in EMG responses at 40 ~ 100 mmHg GD pressure compared to the NS group (Figure 2A,B, *n* = 6 rats for each group, *p < 0.05, **p < 0.01, ***p < 0.001 compared to PMS + NS, two‐way repeated‐measures ANOVA). These data suggested that FA alleviates GHS. Moreover, FA treatment reversed DNMT1 expression that was reduced by PMS (Figure 2C, *n* = 4 and 7 rats for NS and FA group, respectively, ***p < 0.001, compared to PMS + NS, two‐sample *t*‐test).

### 3.3 FA treatment reverses hyperexcitability of DRG neurons induced by PMS

Since FA inhibited PMS‐induced EMG responses, we investigated whether FA affects the excitability of DRG neurons derived from PMS rats. Under current‐clamp conditions, gastric DRG neurons of PMS rats treated with NS or FA were recorded. Resting potentials (RPs) were –46.1 ± 0.9 and –52.7 ± 1.0 mV for the NS‐ and FA‐treated groups, respectively. Therefore, FA treatment significantly hyperpolarized RPs (Figure 2D, *n* = 17 and 13 cells from NS‐ and FA‐treated groups, respectively, ***p < 0.001 compared to NS, two‐sample *t*‐test). The rheobase measurements were 0.014 ± 0.002 and 0.024 ± 0.004 nA for the NS‐ and FA‐treated groups, respectively. The administration of FA markedly increased rheobase (Figure 2E, *n* = 17 and 13 cells from NS‐ and FA‐treated groups, respectively, *p < 0.05 compared to NS, two‐sample *t*‐test). The action potential (AP) thresholds were –32.9 ± 0.949 and –27 ± 0.002 mV for NS‐ and FA‐treated groups, respectively. The FA treatment also increased the AP thresholds (Figure 2F, *n* = 17 and 13 cells from NS‐ and FA‐treated groups, respectively, **p < 0.01 compared to NS, two‐sample *t*‐test). FA treatment also greatly decreased the number of APs evoked by 2X and 3X rheobase current stimulation (Figure 2G,H, *n* = 17 and 13 cells from NS and FA‐treated groups, respectively, *p < 0.05 compared to NS, two‐sample *t*‐test). The numbers of AP evoked by 2X rheobase current stimulation were 2.3 ± 0.2 and 1.6 ± 0.2 for NS‐ and FA‐treated groups, respectively. The numbers of AP evoked by 3X rheobase current stimulation were 3.2 ± 0.4 and 2.2 ± 0.2 for NS‐ and FA‐treated groups, respectively. In addition, FA treatment greatly reduced the number of APs evoked by 100, 300, and 500 pA ramp current stimulation. The numbers of APs evoked by 100 pA ramp current stimulation were 13.8 ± 2.2 and 7.0 ± 1.5 for the NS‐ and FA‐treated groups, respectively. The numbers of APs evoked by 300 pA ramp current stimulation were 17.1 ± 2.5 and 9.8 ± 1.8 for the NS‐ and FA‐treated groups, respectively. The numbers of APs evoked by 500 pA ramp current stimulation were 21.2 ± 2.8 and 12.1 ± 2.2 for the NS‐ and FA‐treated groups, respectively (Figure 2I,J, *n* = 17 and 13 cells from NS‐ and FA‐treated groups, respectively, *p < 0.05 compared to NS, two‐sample *t*‐test).

### 3.4 FA suppresses ASICs hypersensitivity of DRG neurons induced by PMS

**FIGURE 3:**
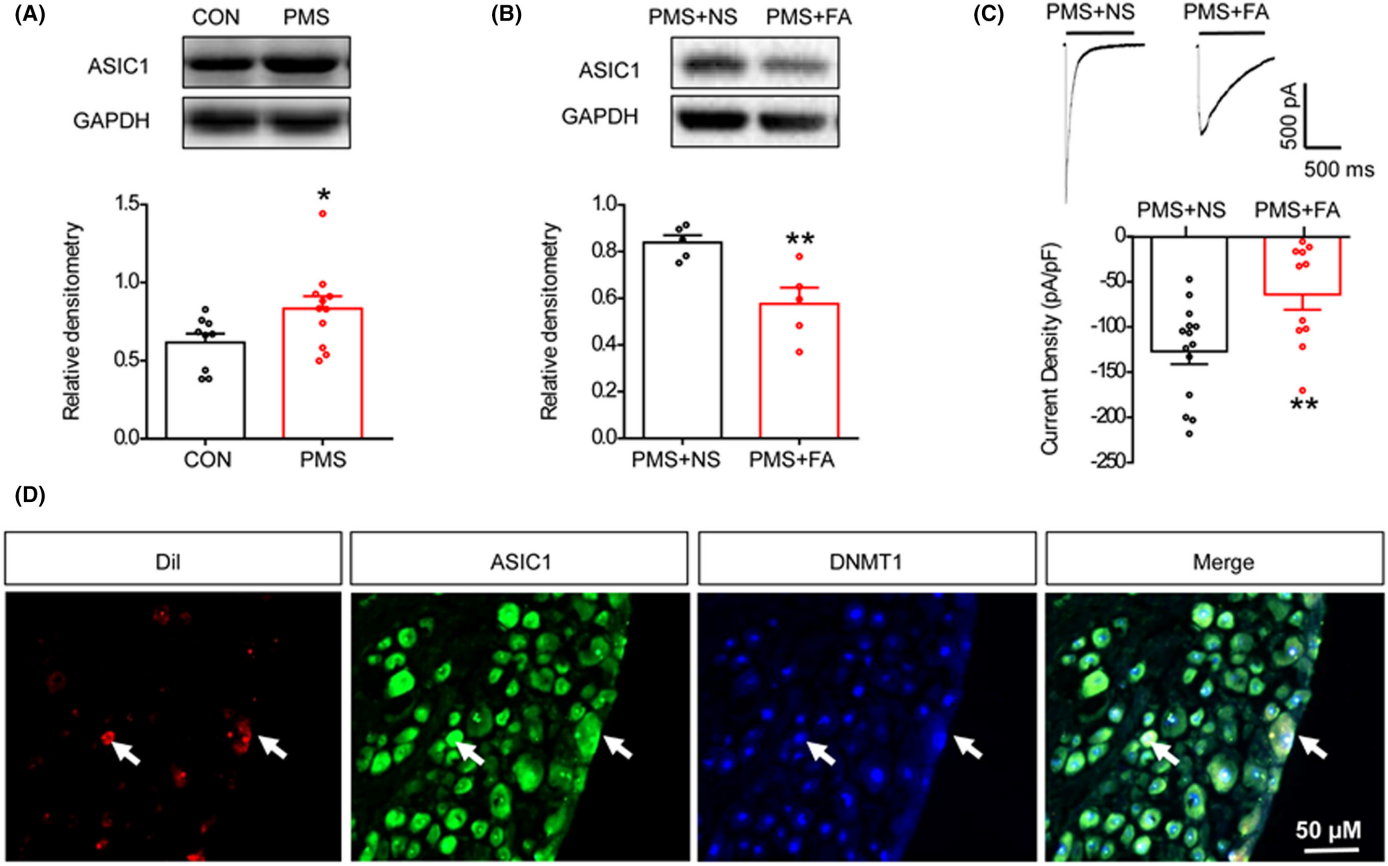
Suppression of ASIC1 by FA in DRG neurons. (A) Western blot assays demonstrated ASIC1 protein expression of T7–T10 DRGs that was increased in PMS rats (*n* = 9 and 11 rats for CON and PMS group, respectively, *p < 0.05 vs. CON, two‐sample *t*‐test). (B) Administration of FA significantly decreased ASIC1 protein expression of T7–T10 DRGs in PMS rats (*n* = 5 rats for each group, **p < 0.01 vs. NS, two‐sample *t*‐test). (C) Treatment of FA significantly decreased the average peak current density of recorded neurons in response to a pH 6.0 (NS, *n* = 14 cells; FA, *n* = 11 cells, **p < 0.01 vs. NS, two‐sample *t*‐test). (D) Gastric‐specific DRG neurons were labeled with DiI (red), which was injected into the stomach wall. ASIC1‐ and DNMT1‐positive neurons were shown in green and blue (scale bar 50 μm). ASIC1 and DNMT1 were co‐localized in gastric‐specific DRG neurons.

ASICs are the main H^+^ receptors in the nervous system and ASIC1, the major subunit responsible for acid‐activated current, plays pivotal roles in diverse functions including synaptic transmission and plasticity.^37^ We have shown that ASIC1 contributes to PMS‐induced GHS.^8^ In the present study, PMS increased ASIC1 expression (Figure 3A, *n* = 9 and 11 rats for CON and PMS group, respectively, *p < 0.05 compared to CON, two‐sample *t*‐test) and FA treatment significantly decreased ASIC1 in PMS rats (Figure 3B, *n* = 5 rats for each group, **p < 0.01 compared to NS, two‐sample *t*‐test). In addition, currents evoked by H^+^ in DRG neurons obtained from NS‐ and FA‐treated PMS rats were measured. At 260 mV holding potential, the recorded neuron expressed a transient inward current in response to pH 6.0. The average peak current density was −127.0 ± 14.2 and –64.0 ± 16.8 pA/pF for the NS and FA groups, respectively (Figure 3C, *n* = 14 and 11 cells from NS‐ and FA‐treated groups, respectively, **p < 0.01 compared to NS, two‐sample *t*‐test). Therefore, FA could suppress the ASIC sensitivity activated by PMS. Furthermore, DiI was injected into the stomach wall to label gastric DRG neurons, followed by double immunofluorescence using ASIC1 and DMNT1 antibodies. The results showed that ASIC1 and DNMT1 were co‐localized in gastric DRG neurons (Figure 3D). This suggested a possibility of the interaction between DNMT1 and ASIC1 in PMS‐induced GHS.

### 3.5 DNMT1 inhibitor DC‐517 reverses the analgesic effect of FA on GHS

**FIGURE 4:**
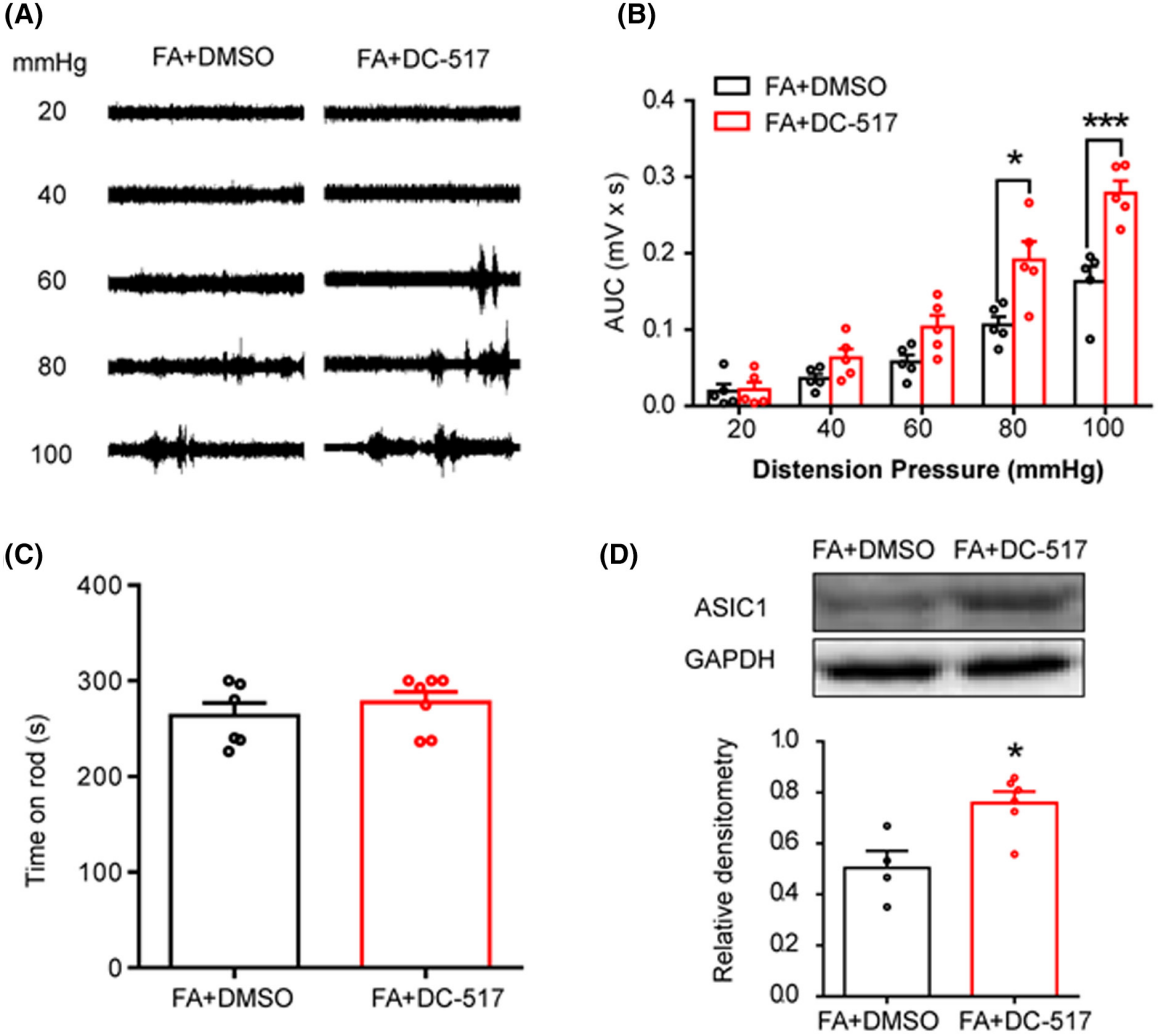
Administration of DNMT1 inhibitor DC‐517 reverses analgesic effect of FA on GHS. (A) Representative EMG recordings from FA + DMSO and FA + DC‐517 rats responding to graded GD at 20, 40, 60, 80, and 100 mmHg in PMS rats. (B) FA + DC‐517 at the dose of 100 μM in 10 μL dramatically increased the AUC value at 80 and 100 mmHg GD pressures (*n* = 5 rats for each group, *p < 0.05, ***p < 0.001 vs. FA + DMSO, two‐way repeated‐measures ANOVA). (C) DC‐517 treatment did not affect the time for rats to stay on the rod compared to FA + DMSO treatment in PMS rats (*n* = 6 and 7 rats for DMSO and DC‐517 group, respectively, *p* > 0.05 vs. FA + DMSO, two‐sample *t*‐test). (D) Western blot results showed that DC‐517 at the dose of 100 μM in 10 μL reverses ASIC1 protein expression in T7‐T10 DRGs of PMS rats (*n* = 4 and 6 rats for DMSO and DC‐517 group, respectively, *p < 0.05 vs. FA + DMSO, two‐sample *t*‐test).

To assess the role of DNMT1 in the analgesic effect of FA on GHS, we first investigated the effect of DC‐517, a specific DNMT1 inhibitor, on EMG amplitude in response to GD. At the age of 6 weeks, FA‐treated PMS rats received an intrathecal injection of DC‐517 (100 μM in 10 μL) or DMSO once a day for 7 consecutive days. The results showed that significant effects were observed at distension pressures of 80 and 100 mmHg in the DC‐517‐treated group (Figure 4A,B, *n* = 5 rats for each group, *p < 0.05, ***p < 0.001, compared to FA + DMSO, two‐way repeated‐measures ANOVA). The motor function was not affected (Figure 4C, *n* = 6 and 7 rats for DMSO and DC‐517 group, respectively, *p* > 0.05, compared to FA + DMSO, two‐sample *t*‐test). These findings suggested that DC‐517 inhibited the analgesic effect of FA on GHS. Moreover, DC‐517 reversed ASIC1 expression reduced by FA (Figure 4D, *n* = 4 and 6 rats for DMSO and DC‐517 group, respectively, *p < 0.05 compared to FA + DMSO, two‐sample *t*‐test).

### 3.6 DNMT1 overexpression suppresses GHS and ASIC1 hypersensitivity

**FIGURE 5:**
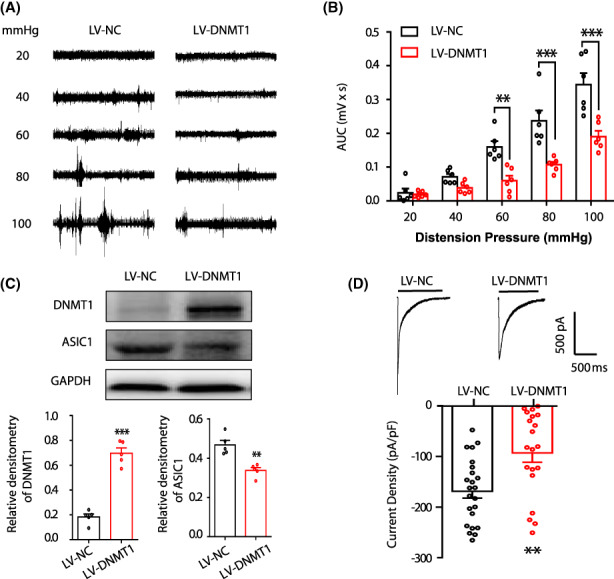
DNMT1 overexpression suppresses GHS and ASIC1 sensitivity. (A) Representative EMG recordings from LV‐NC and LV‐DNMT1 rats responding to graded GD at 20, 40, 60, 80, and 100 mmHg in PMS rats. (B) DNMT1 overexpression significantly decreased the AUC value at 60, 80, and 100 mmHg GD pressures (*n* = 6 rats for each group, **p < 0.01, ***p < 0.001 vs. LV‐NC, two‐way repeated‐measures ANOVA). (C) DNMT1 overexpression increased DNMT1 protein expression in T7–10 DRGs of PMS rats (*n* = 5 rats for each group, ***p < 0.001 vs. LV‐NC, two‐sample *t*‐test) and decreased ASIC1 protein expression in T7–10 DRGs of PMS rats (*n* = 5 rats for each group, **p < 0.05 vs. LV‐NC, two‐sample *t*‐test). The same GAPDH bands were used as loading controls for relative densitometry analyses of DNMT1 and ASIC1. (D) Overexpression of DNMT1 decreased the average peak current density of recorded neurons in response to a pH 6.0 (LV‐NC, *n* = 23 cells; LV‐DNMT1, *n* = 20 cells, **p < 0.01 vs. LV‐NC, two‐sample *t*‐test).

To examine whether DNMT1 contributes to PMS‐induced GHS and ASIC1 hypersensitivity, LV‐NC or LV‐DNMT1 was intrathecally injected in PMS rats at the age of 5 weeks. Two weeks after injection, EMG was recorded. LV‐NC treatment had no significant effect on EMG amplitude. However, LV‐DNMT1 resulted in a dramatic reduction in EMG responses at 60, 80, and 100 mmHg GD pressures, as evident by the AUC (Figure 5A,B, *n* = 6 rats for each group, **p < 0.01, ***p < 0.001 compared to LV‐NC, two‐way repeated‐measures ANOVA). These data suggested that DNMT1 was involved in the development of PMS‐induced GHS. To further determine the correlation between DNMT1 and ASIC1, we examined whether LV‐DNMT1 suppresses ASIC1 expression and sensitivity. Western blot analysis showed that LV‐DNMT1 treatment significantly reversed DNMT1 expression that was downregulated by PMS (Figure 5C, *n* = 5 rats for each group, ***p < 0.001, compared to LV‐NC, two‐sample *t*‐test). Furthermore, LV‐DNMT1, significantly blocked the increase of ASIC1 induced by PMS (Figure 5C, *n* = 5 rats for each group, **p < 0.01, compared to LV‐NC, two‐sample *t*‐test). In addition, currents evoked by H^+^ in DRG neurons obtained from LV‐NC‐ or LV‐DNMT1‐treated PMS rats were measured. The average peak current density was −168.8 ± 13.8 and −93.1 ± 18.5 pA/pF for the LV‐NC and LV‐DNMT1 groups, respectively (Figure 5D, *n* = 23 and 20 cells from LV‐NC and LV‐DNMT1 groups, respectively, **p < 0.01 compared to LV‐NC, two‐sample *t*‐test). Together, these findings indicated that DNMT1 suppressed ASIC1 expression and hypersensitivity induced by PMS.

### 3.7 DNMT1 overexpression decreases neuronal excitability in PMS rats

**FIGURE 6:**
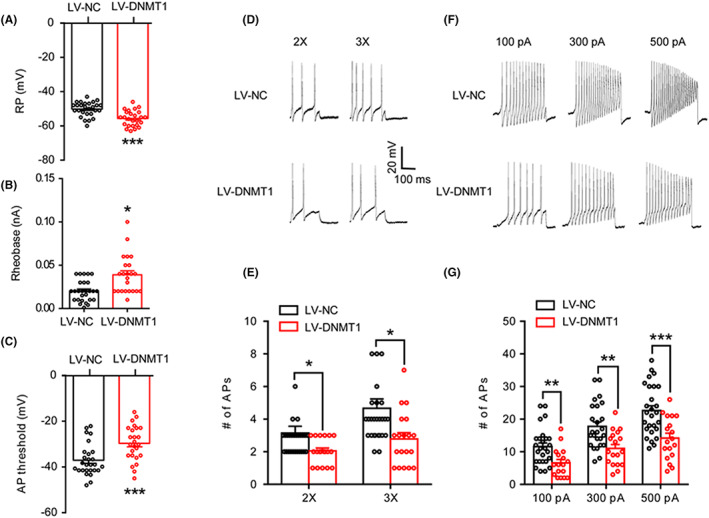
Overexpression of DNMT1 decreases neuronal excitability. (A) Administration of LV‐DNMT1 markedly hyperpolarized RPs (LV‐NC, *n* = 27 cells; LV‐DNMT1, *n* = 25 cells, ***p < 0.001 vs. LV‐NC, two‐sample *t*‐test). (B) Administration of LV‐DNMT1 significantly increased rheobase (LV‐NC, *n* = 27 cells; LV‐DNMT1, *n* = 23 cells, *p < 0.05 vs. LV‐NC, two‐sample *t*‐test). (C) Administration of LV‐DNMT1 remarkably increased AP thresholds (LV‐NC, *n* = 27 cells; LV‐DNMT1, *n* = 25 cells, ***p < 0.001 vs. LV‐NC, two‐sample *t*‐test). (D) Examples of APs by 2X and 3X rheobase current stimulation from LV‐NC (upper) and LV‐DNMT1‐treated (bottom) rats. (E) Bar graph showed a significant decrease in the numbers of APs evoked by 2X and 3X rheobase current stimulation in LV‐DNMT1‐treated rats compared to LV‐NC‐treated rats (LV‐NC, *n* = 27 cells; LV‐DNMT1, *n* = 19 cells, *p < 0.05, two‐sample *t*‐test). (F) Examples of APs by 100, 300, and 500 pA ramp current stimulation from LV‐NC (upper) and LV‐DNMT1‐treated (bottom) rats. (G) Bar graph showed a significant decrease in the numbers of APs evoked by 100, 300, and 500 pA ramp current stimulation in LV‐DNMT1‐treated rats compared to LV‐NC‐treated rats (LV‐NC, *n* = 26, 25, and 27 cells for 100, 300, and 500 pA; LV‐DNMT1, *n* = 17, 18, and 18 cells for 100, 300 and 500 pA, **p < 0.01, ***p < 0.001 vs. LV‐NC, two‐sample *t*‐test).

Since LV‐DNMT1 inhibited EMG responses, we investigated whether LV‐DNMT1 affects the excitability of DRG neurons of PMS rats. Under current‐clamp conditions, DRG neurons derived from LV‐NC‐ or LV‐DNMT1‐treated PMS rats were measured. The RPs were −50.2 ± 0.8 and −55.7 ± 0.9 mV for the LV‐NC and LV‐DNMT1 groups, respectively. Therefore, LV‐DNMT1 treatment significantly hyperpolarized RPs (Figure 6A, *n* = 27 and 25 cells from LV‐NC and LV‐DNMT1 groups, respectively, ***p < 0.001 compared to LV‐NC, two‐sample *t*‐test). The rheobase measurements were 0.02 ± 0.002 and 0.04 ± 0.005 nA for the LV‐NC and LV‐DNMT1 groups, respectively. Administration of LV‐DNMT1 markedly increased rheobase (Figure 6B, *n* = 27 and 23 cells from LV‐NC and LV‐DNMT1 groups, respectively, *p < 0.05 compared to LV‐NC, two‐sample *t*‐test). The AP thresholds were −37.1 ± 0.8 and −29.7 ± 1.5 mV for LV‐NC and LV‐DNMT1 groups, respectively. LV‐DNMT1 treatment also increased the AP thresholds (Figure 6C, *n* = 27 and 25 cells from LV‐NC and LV‐DNMT1 groups, respectively, ***p < 0.001 compared to LV‐NC, two‐sample *t*‐test). LV‐DNMT1 treatment also greatly decreased the number of APs evoked by 2X and 3X rheobase current stimulation (Figure 6D,E, *n* = 27 and 19 cells from LV‐NC and LV‐DNMT1 groups, respectively, *p < 0.05 compared to LV‐NC, two‐sample *t*‐test). The numbers of AP evoked by 2X rheobase current stimulation were 3.1 ± 0.5 and 2.1 ± 0.2 for LV‐NC and LV‐DNMT1 groups, respectively. The numbers of AP evoked by 3X rheobase current stimulation were 4.7 ± 0.6 and 2.8 ± 0.4 for LV‐NC and LV‐DNMT1 groups, respectively. In addition, LV‐DNMT1 treatment greatly reduced the number of APs evoked by 100, 300, and 500 pA ramp current stimulation. The numbers of APs evoked by 100 pA ramp current stimulation were 11.6 ± 1.1 (*n* = 26) and 6.6 ± 1.1 (*n* = 17) for the LV‐NC and LV‐DNMT1 groups, respectively. The numbers of APs evoked by 300 pA ramp current stimulation were 17.8 ± 1.4 (*n* = 25) and 11 ± 1.3 (*n* = 18) for the LV‐NC and LV‐DNMT1 groups, respectively. The numbers of APs evoked by 500 pA ramp current stimulation were 22.6 ± 1.5 (*n* = 27) and 14.2 ± 1.5 (*n* = 18) for the LV‐NC and LV‐DNMT1 groups, respectively (Figures 6F,G, **p < 0.01 and ***p < 0.001 compared to LV‐NC, two‐sample *t*‐test).

## 4 DISCUSSION

We have reported that PMS induced GHS in adult offspring, accompanied by DNMT1 decrease and ASIC1 increase.^8^ The present study showed that FA ameliorates PMS‐induced GHS via increasing DNMT1 and reducing ASIC1 sensitivity and neuronal excitability. More importantly, DNMT1 inhibitor DC‐517 blocked the analgesic effect of FA, and DNMT1 overexpression reduced neuronal hyperexcitability and GHS induced by PMS.

Environmental factors that induce methylation of gene promoters might regulate disease progression.^38^ DNA methylation is regulated by DNMT^39^ and demethylase.^40^, ^41^ In the present study, DNMT1 expression, but not DNMT3a and DNMT3b, was decreased in PMS rats. This phenomenon was consistent with our previous report that DNMTs were downregulated under diabetic neuropathic pain and bone cancer pain.^32^, ^42^, ^43^ However, it was different from another study that showed an active mechanism in inflammatory pain.^44^ Although the detailed mechanism for this difference is unclear, the role of DNMT1 in PMS‐induced GHS may differ from that of inflammatory and neuropathic pain.

According to the clinical phenotype, pregnant women need to supplement FA as it can prevent various malformations of neural tubes.^45^, ^46^, ^47^ FA also induces spinal axon regeneration via DNA methylation^26^, ^48^, ^49^ and alleviates neuropathic pain in spinal cord injury rats.^50^ However, the role of FA in PMS‐induced GHS was unclear. The present study showed that FA alleviated GHS, accompanied by increased DNMT1 expression and decreased neuronal excitability. Moreover, PMS upregulated ASIC1 expression that was in accordance with our previous report.^8^ FA significantly decreased ASIC1 expression and sensitivity. These findings indicated that FA might be a potential therapeutic target for FD.

Next, the mechanism of analgesic effect of FA on GHS was further explored. Double immunofluorescence results showed that DNMT1 and ASIC1 were co‐localized in DRG neurons, suggesting a possibility of the interaction between DNMT1 and ASIC1. Treatment with DNMT1 inhibitor DC‐517 not only blocked the analgesic effect of FA, but also increased ASIC1 expression. These results further supported that DNMT1 mediated the analgesic effect of FA on GHS by regulating ASIC1. Whether additional molecules are involved in DNMT1 conducting FA analgesic effect remains to be investigated. Furthermore, overexpression of DNMT1 by LV‐DNMT1 not only alleviates GHS and ASIC1 hypersensitivity but also decreased neuronal excitability. These findings suggested that DNMT1 might be a potential target of GHS induced by PMS, but detailed mechanisms of how DNMT1 work to regulate and interact with ASIC1 remains to be addressed in future studies.

Recent studies have shown that sex dimorphism occurs on all expects of brain perfusion, cerebrovascular functions, and activities of neuro circuits.^51^, ^52^ Furthermore, chronic prenatal stress upregulated brain‐derived neurotrophic factors that correlated with the exacerbation of visceral hypersensitivity in female, but not in male offspring.^53^ So, the mechanism of analgesic effect of FA on PMS‐induced GHS in females might be different from male offspring. It is of great significance to include both sexes of animals in our future study.

In conclusion, our study showed that inhibition of DNMT1 blocked analgesic effect of FA on GHS and reversed ASIC1 expression that was decreased by FA. Moreover, DNMT1 overexpression alleviated GHS and inhibited ASIC1 expression and sensitivity. These findings suggest that DNMT1 is involved in the analgesic effect of FA on PMS‐induced GHS by regulating ASIC1. It proposes that DNMT1 may be a potential target for visceral pain management.

## AUTHOR CONTRIBUTIONS

H‐J. W performed experiments, analyzed data, and prepared the manuscript. F‐C. Z and T‐W. X performed experiments and analyzed data. Y‐C. X performed experiments, analyzed data and prepared the figures. Y‐Q. T and Y‐Y. W performed experiments and analyzed data. J‐T X and S. H analyzed data and prepared the manuscript. G‐Y. X designed experiments, supervised the experiments, and finalized the manuscript. All the authors have read and approved the paper.

## CONFLICT OF INTEREST STATEMENT

No conflicts of interest, financial or otherwise, are declared by the authors.

## Data Availability

The data that support the findings of this study are available from the corresponding author upon reasonable request.

## References

[1] Stanghellini V , Chan FK , Hasler WL , et al. Gastroduodenal Disorders. Gastroenterology. 2016;150(6):1380‐1392. 27147122 10.1053/j.gastro.2016.02.011

[2] Tack J , Talley NJ , Camilleri M , et al. Functional gastroduodenal disorders. Gastroenterology. 2006;130(5):1466‐1479. 16678560 10.1053/j.gastro.2005.11.059

[3] Tack J , Caenepeel P , Fischler B , Piessevaux H , Janssens J . Symptoms associated with hypersensitivity to gastric distention in functional dyspepsia. Gastroenterology. 2001;121(3):526‐535. 11522735 10.1053/gast.2001.27180

[4] Miwa H , Watari J , Fukui H , et al. Current understanding of pathogenesis of functional dyspepsia. J Gastroenterol Hepatol. 2011;26(Suppl 3):53‐60. 21443711 10.1111/j.1440-1746.2011.06633.x

[5] Keohane J , Quigley EM . Functional dyspepsia: the role of visceral hypersensitivity in its pathogenesis. World J Gastroenterol. 2006;12(17):2672‐2676. 16718751 10.3748/wjg.v12.i17.2672 PMC4130973

[6] Winston JH , Sarna SK . Developmental origins of functional dyspepsia‐like gastric hypersensitivity in rats. Gastroenterology. 2013;144(3):570‐579 e573. 23142231 10.1053/j.gastro.2012.11.001 PMC3578170

[7] Geeraerts B , Van Oudenhove L , Fischler B , et al. Influence of abuse history on gastric sensorimotor function in functional dyspepsia. Neurogastroenterol Motil. 2009;21(1):33‐41. 18694440 10.1111/j.1365-2982.2008.01178.x

[8] Wang HJ , Xu X , Zhang PA , et al. Epigenetic upregulation of acid‐sensing ion channel 1 contributes to gastric hypersensitivity in adult offspring rats with prenatal maternal stress. Pain. 2020;161(5):989‐1004. 31895269 10.1097/j.pain.0000000000001785

[9] Hong S , Zheng G , Wiley JW . Epigenetic regulation of genes that modulate chronic stress‐induced visceral pain in the peripheral nervous system. Gastroenterology. 2015;148(1):148‐157 e147. 25263804 10.1053/j.gastro.2014.09.032 PMC4274248

[10] Cao DY , Bai G , Ji Y , Traub RJ . Epigenetic upregulation of metabotropic glutamate receptor 2 in the spinal cord attenuates oestrogen‐induced visceral hypersensitivity. Gut. 2015;64(12):1913‐1920. 25378524 10.1136/gutjnl-2014-307748 PMC4562903

[11] Higgins GA , Hong S , Wiley JW . The role of epigenomic regulatory pathways in the gut‐brain Axis and visceral hyperalgesia. Cell Mol Neurobiol. 2022;42(2):361‐376. 34057682 10.1007/s10571-021-01108-0 PMC10286693

[12] Okano M , Bell DW , Haber DA , Li E . DNA methyltransferases Dnmt3a and Dnmt3b are essential for de novo methylation and mammalian development. Cell. 1999;99(3):247‐257. 10555141 10.1016/s0092-8674(00)81656-6

[13] Liang L , Lutz BM , Bekker A , Tao YX . Epigenetic regulation of chronic pain. Epigenomics. 2015;7(2):235‐245. 25942533 10.2217/epi.14.75 PMC4422180

[14] Ostuni R , Piccolo V , Barozzi I , et al. Latent enhancers activated by stimulation in differentiated cells. Cell. 2013;152(1–2):157‐171. 23332752 10.1016/j.cell.2012.12.018

[15] Domcke S , Bardet AF , Adrian Ginno P , Hartl D , Burger L , Schubeler D . Competition between DNA methylation and transcription factors determines binding of NRF1. Nature. 2015;528(7583):575‐579. 26675734 10.1038/nature16462

[16] Sun L , Gu X , Pan Z , et al. Contribution of DNMT1 to neuropathic pain genesis partially through epigenetically repressing Kcna2 in primary afferent neurons. J Neurosci. 2019;39(33):6595‐6607. 31182635 10.1523/JNEUROSCI.0695-19.2019 PMC6697395

[17] Yuan J , Wen J , Wu S , et al. Contribution of dorsal root ganglion octamer transcription factor 1 to neuropathic pain after peripheral nerve injury. Pain. 2019;160(2):375‐384. 30247265 10.1097/j.pain.0000000000001405 PMC6344274

[18] Zhao JY , Liang L , Gu X , et al. DNA methyltransferase DNMT3a contributes to neuropathic pain by repressing Kcna2 in primary afferent neurons. Nat Commun. 2017;8:14712. 28270689 10.1038/ncomms14712 PMC5344974

[19] Smith AD , Smith SM , de Jager CA , et al. Homocysteine‐lowering by B vitamins slows the rate of accelerated brain atrophy in mild cognitive impairment: a randomized controlled trial. PLoS One. 2010;5(9):e12244. 20838622 10.1371/journal.pone.0012244 PMC2935890

[20] Faux NG , Ellis KA , Porter L , et al. Homocysteine, vitamin B12, and folic acid levels in Alzheimer's disease, mild cognitive impairment, and healthy elderly: baseline characteristics in subjects of the Australian imaging biomarker lifestyle study. J Alzheimers Dis. 2011;27(4):909‐922. 21891867 10.3233/JAD-2011-110752

[21] Zhou Q , Gensch C , Liao JK . Rho‐associated coiled‐coil‐forming kinases (ROCKs): potential targets for the treatment of atherosclerosis and vascular disease. Trends Pharmacol Sci. 2011;32(3):167‐173. 21242007 10.1016/j.tips.2010.12.006 PMC3080120

[22] Kronenberg G , Colla M , Endres M . Folic acid, neurodegenerative and neuropsychiatric disease. Curr Mol Med. 2009;9(3):315‐323. 19355913 10.2174/156652409787847146

[23] Greene ND , Copp AJ . Neural tube defects. Annu Rev Neurosci. 2014;37:221‐242. 25032496 10.1146/annurev-neuro-062012-170354 PMC4486472

[24] Castillo‐Lancellotti C , Tur JA , Uauy R . Impact of folic acid fortification of flour on neural tube defects: a systematic review. Public Health Nutr. 2013;16(5):901‐911. 22850218 10.1017/S1368980012003576 PMC10271422

[25] De‐Regil LM , Pena‐Rosas JP , Fernandez‐Gaxiola AC , Rayco‐Solon P . Effects and safety of periconceptional oral folate supplementation for preventing birth defects. Cochrane Database Syst Rev. 2015;2015(12):CD007950. 26662928 10.1002/14651858.CD007950.pub3 PMC8783750

[26] Iskandar BJ , Rizk E , Meier B , et al. Folate regulation of axonal regeneration in the rodent central nervous system through DNA methylation. J Clin Invest. 2010;120(5):1603‐1616. 20424322 10.1172/JCI40000 PMC2860927

[27] Luo S , Zhang X , Yu M , et al. Folic acid acts through DNA methyltransferases to induce the differentiation of neural stem cells into neurons. Cell Biochem Biophys. 2013;66(3):559‐566. 23292356 10.1007/s12013-012-9503-6

[28] Piyathilake CJ , Celedonio JE , Macaluso M , Bell WC , Azrad M , Grizzle WE . Mandatory fortification with folic acid in the United States is associated with increased expression of DNA methyltransferase‐1 in the cervix. Nutrition. 2008;24(1):94‐99. 18065205 10.1016/j.nut.2007.10.007 PMC2215322

[29] Sayem ASM , Giribabu N , Muniandy S , Salleh N . Effects of thyroxine on expression of proteins related to thyroid hormone functions (TR‐alpha, TR‐beta, RXR and ERK1/2) in uterus during peri‐implantation period. Biomed Pharmacother. 2017;96:1016‐1021. 29221723 10.1016/j.biopha.2017.11.128

[30] Wang HJ , Xu X , Xie RH , et al. Prenatal maternal stress induces visceral hypersensitivity of adult rat offspring through activation of cystathionine‐beta‐synthase signaling in primary sensory neurons. Mol Pain. 2018;14:1744806918777406. 29712513 10.1177/1744806918777406 PMC5967159

[31] Winston JH , Xu GY , Sarna SK . Adrenergic stimulation mediates visceral hypersensitivity to colorectal distension following heterotypic chronic stress. Gastroenterology. 2010;138(1):294‐304 e293. 19800336 10.1053/j.gastro.2009.09.054 PMC2813397

[32] Zhang HH , Hu J , Zhou YL , et al. Promoted interaction of nuclear factor‐kappaB with demethylated purinergic P2X3 receptor gene contributes to neuropathic pain in rats with diabetes. Diabetes. 2015;64(12):4272‐4284. 26130762 10.2337/db15-0138

[33] Xu GY , Li G , Liu N , Huang LY . Mechanisms underlying purinergic P2X3 receptor‐mediated mechanical allodynia induced in diabetic rats. Mol Pain. 2011;7:60. 21851615 10.1186/1744-8069-7-60 PMC3168406

[34] Xu GY , Huang LY . Peripheral inflammation sensitizes P2X receptor‐mediated responses in rat dorsal root ganglion neurons. J Neurosci. 2002;22(1):93‐102. 11756492 10.1523/JNEUROSCI.22-01-00093.2002 PMC6757597

[35] Sun Y , Yang PP , Song ZY , et al. Alpha‐lipoic acid suppresses neuronal excitability and attenuates colonic hypersensitivity to colorectal distention in diabetic rats. J Pain Res. 2017;10:1645‐1655. 28769585 10.2147/JPR.S135017 PMC5529097

[36] Rozas G , Labandeira Garcia JL . Drug‐free evaluation of rat models of parkinsonism and nigral grafts using a new automated rotarod test. Brain Res. 1997;749(2):188‐199. 9138718 10.1016/S0006-8993(96)01162-6

[37] Song XL , Liu DS , Qiang M , et al. Postsynaptic targeting and mobility of membrane surface‐localized hASIC1a. Neurosci Bull. 2021;37(2):145‐165. 32996060 10.1007/s12264-020-00581-9 PMC7870742

[38] Fuso A , Scarpa S . One‐carbon metabolism and Alzheimer's disease: is it all a methylation matter? Neurobiol Aging. 2011;32(7):1192‐1195. 21524430 10.1016/j.neurobiolaging.2011.01.012

[39] Lyko F . The DNA methyltransferase family: a versatile toolkit for epigenetic regulation. Nat Rev Genet. 2018;19(2):81‐92. 29033456 10.1038/nrg.2017.80

[40] Wang SD , Wang X , Zhao Y , et al. Homocysteine‐induced disturbances in DNA methylation contribute to development of stress‐associated cognitive decline in rats. Neurosci Bull. 2022;38(8):887‐900. 35435568 10.1007/s12264-022-00852-7 PMC9352847

[41] Ghazimoradi MM , Ghorbani MH , Ebadian E , et al. Epigenetic effects of graphene oxide and its derivatives: a mini‐review. Mutat Res Genet Toxicol Environ Mutagen. 2022;878:503483. 35649677 10.1016/j.mrgentox.2022.503483

[42] Zhou YL , Jiang GQ , Wei J , et al. Enhanced binding capability of nuclear factor‐kappaB with demethylated P2X3 receptor gene contributes to cancer pain in rats. Pain. 2015;156(10):1892‐1905. 26049406 10.1097/j.pain.0000000000000248 PMC4770335

[43] Wu YY , Zhang HL , Lu X , et al. Targeting GATA1 and p2x7r locus binding in spinal astrocytes suppresses chronic visceral pain by promoting DNA demethylation. Neurosci Bull. 2022;38(4):359‐372. 34890016 10.1007/s12264-021-00799-1 PMC9068853

[44] Li F , Xue ZY , Yuan Y , et al. Upregulation of CXCR4 through promoter demethylation contributes to inflammatory hyperalgesia in rats. CNS Neurosci Ther. 2018;24(10):947‐956. 29577638 10.1111/cns.12845 PMC6489799

[45] Shaw GM , Todoroff K , Carmichael SL , Schaffer DM , Selvin S . Lowered weight gain during pregnancy and risk of neural tube defects among offspring. Int J Epidemiol. 2001;30(1):60‐65. 11171858 10.1093/ije/30.1.60

[46] Scholl TO , Johnson WG . Folic acid: influence on the outcome of pregnancy. Am J Clin Nutr. 2000;71(5 Suppl):1295 S‐1303 S. 10.1093/ajcn/71.5.1295s 10799405

[47] Guo J , Ni S , Li Q , Wang JZ , Yang Y . Folate/vitamin B alleviates Hyperhomocysteinemia‐induced Alzheimer‐like pathologies in rat retina. Neurosci Bull. 2019;35(2):325‐335. 30264378 10.1007/s12264-018-0293-8 PMC6426902

[48] Iskandar BJ , Nelson A , Resnick D , et al. Folic acid supplementation enhances repair of the adult central nervous system. Ann Neurol. 2004;56(2):221‐227. 15293274 10.1002/ana.20174

[49] Shi J , Sun S , Wang Y , Huang Z . Reprogramming restores vision in mice by changing DNA methylation. Neurosci Bull. 2021;37(10):1526‐1528. 34097224 10.1007/s12264-021-00729-1 PMC8490542

[50] Miranpuri GS , Meethal SV , Sampene E , et al. Folic acid modulates matrix Metalloproteinase‐2 expression, alleviates neuropathic pain, and improves functional recovery in spinal cord‐injured rats. Ann Neurosci. 2017;24(2):74‐81. 28588362 10.1159/000475896 PMC5448437

[51] Wang R , Oh JM , Motovylyak A , et al. Impact of sex and APOE ε4 on age‐related cerebral perfusion trajectories in cognitively asymptomatic middle‐aged and older adults: a longitudinal study. J Cereb Blood Flow Metab. 2021;41(11):3016‐3027. 34102919 10.1177/0271678X211021313 PMC8545048

[52] Chandra PK , Cikic S , Baddoo MC , et al. Transcriptome analysis reveals sexual disparities in gene expression in rat brain microvessels. J Cereb Blood Flow Metab. 2021;41(9):2311‐2328. 33715494 10.1177/0271678X21999553 PMC8392780

[53] Winston JH , Li Q , Sarna SK . Chronic prenatal stress epigenetically modifies spinal cord BDNF expression to induce sex‐specific visceral hypersensitivity in offspring. Neurogastroenterol Motil. 2014;26(5):715‐730. 24588943 10.1111/nmo.12326 PMC3997587

